# Acute effects of N‐terminal progastrin fragments on gastric acid secretion in man

**DOI:** 10.14814/phy2.13164

**Published:** 2017-03-08

**Authors:** Jens P. Goetze, Carsten P. Hansen, Jens F. Rehfeld

**Affiliations:** ^1^ Department of Clinical BiochemistryRigshospitaletUniversity of CopenhagenCopenhagenDenmark; ^2^ Department of Gastrointestinal SurgeryRigshospitaletUniversity of CopenhagenCopenhagenDenmark

**Keywords:** Acid secretion, inhibition, N‐terminal, progastrin, regulation

## Abstract

We previously identified an N‐terminal fragment of progastrin in human antrum and plasma, where it circulates in high concentrations. In this study, we examined the effects of N‐terminal progastrin fragments on gastric acid secretion by infusion in healthy individuals. Increasing doses of progastrin fragment 1‐35 were infused intravenously during constant gastric acid stimulation by gastrin‐17. In addition, the effects of progastrin fragment 1‐35, fragment 6‐35, and fragment 1‐19 on gastrin‐17 stimulated acid secretion were tested. The gastrin‐17 stimulated acid secretion decreased 30% after administration of a high dose of progastrin fragment 1‐35 (*P* < 0.05). In extension, a 1‐h infusion of fragment 1‐35 also decreased gastric acid output. In contrast, fragment 6‐35 did not affect acid secretion, and a single infusion of gastrin‐17 alone did not reveal fading of gastric acid output during the time course of the experiments. The results show that N‐terminal fragments of progastrin may acutely affect gastrin‐stimulated gastric acid secretion in vivo. Structure‐function analysis suggests that the N‐terminal pentapeptide of progastrin is required for the effect.

## Introduction

The gastrointestinal hormone gastrin is the major regulator of gastric acid secretion (Gregory and Tracy 1964). Gastrin is synthesized in antroduodenal G‐cells by elaborate processing of progastrin into a number of acid stimulatory peptides (Gregory 1974), of which gastrin‐34 and gastrin‐17 predominate in circulation (Yalow and Berson 1970; Rehfeld 1972). Common for all acid stimulatory gastrins is the C‐terminal active site, Trp‐Met‐Asp‐Phe‐NH_2_ (Morley et al. 1965; Gregory 1974).

In addition to acid stimulatory gastrins, complementary progastrin‐derived fragments are also released from G‐cells. N‐terminal progastrin fragments have been identified in gastrinoma tissue (Reeve et al. 1984; Desmond et al. 1987; Huebner et al. 1991) and non‐neoplastic human antral tissue (Rehfeld and Johnsen 1994). Besides intact progastrin 1‐80, the N‐terminal fragments 1‐35, 6‐35, and 20‐36 were identified by mass spectrometry, the 1‐35 fragment being the most abundant. We have also shown that N‐terminal fragments circulate in almost 30‐fold higher concentration than gastrins (286 versus 10 pmol/L) and with a long elimination phase as compared to gastrin‐17 (Goetze et al. 2006). Besides gastrins, dominately gastrin‐17 and gastrin 34, and N‐terminal prograstrin fragments, dominately progastrin 1‐35 and prograstrin 1‐19, a mixture of other processing‐intermediates, namely glycine‐extended gastrins, are circulating; please see Rehfeld 2014 for a recent review. Apart for gastrins, however, no specific receptor has been identified for any of the processing intermediates.

Comparative studies have disclosed that the C‐terminal pentapeptide of bioactive gastrins and the N‐terminal pentapeptide sequence of progastrin are both fully preserved in mammals (Yoo et al. 1982; Boel et al. 1983; Johnsen and Rehfeld 1993). The evolutionary conservation, taken together with the occurrence of fragment 1‐35 in high concentrations in antral tissue and plasma, raise the possibility of a biological function of the N‐terminal fragments. In this study, we examined the effects of these fragments on stimulated gastric acid secretion in healthy individuals.

## Materials and Methods

### Peptides

N‐terminal fragment 1‐35 of human progastrin was custom synthesized (Kem‐En‐Tec, Copenhagen, Denmark), as was fragment 6‐35 and fragment 1‐19 (Cambridge Research Biochemicals Ltd). The identity and purity of all peptides was verified by amino acid analysis (Amino Quant, Hewlett Packard, Waldron, Germany) and mass spectrometry (MALDI‐TOF, Biflex, Bruker, Bremen, Germany). Synthetic human non‐sulfated gastrin‐17 was purchased from Sigma Chemical (St. Louis, MO).

### Subjects

Healthy individuals of both genders were invited for the studies and all were free of symptoms and signs of gastric disease (median age 24, range 22–27 years). No subject included in the study had immunological signs of prior or present *H. Pylori* infection. Written and informed consent was obtained from all the volunteers, and the study was approved by the local ethics committee for medical research in Copenhagen (KF 02‐185/95) in accordance with the Helsinki II declaration.

### Experimental procedures

The infusion studies were carried out randomly on separate days.

#### A) Acid secretion during a constant infusion of gastrin‐17 (*n* = 6)

After an overnight fast, a nasogastric double‐lumen tube (AN10, Andersen Prod. Inc., NC) was inserted with its tip in the antrum under fluoroscopic control. The residual gastric content had been evacuated and then the gastric juice was continuously aspirated and collected by an intermittent‐suction apparatus (Egnell, Sweden) in 15 min intervals. Subjects were positioned in the supine position throughout the examination. After 1 h, gastric acid secretion was stimulated for 45 min with a 20 pmol/kg/h infusion with non‐sulfated gastrin‐17 (hereafter mentioned as gastrin‐17) using a calibrated pump (Perfusor Vll, Braun Melsungen, Melsungen, Germany). After this period, a 40 pmol/kg/h gastrin‐17 infusion was administered during the remaining time of the examination. This dose rate was chosen because we have earlier found it to stimulate gastric acid secretion to approximately 50% of maximal output. Venous blood samples were drawn every 15 min from a cubital vein in the arm not used for infusion. Acid secretion output was evaluated using nonparametric testing for each time point (15 min) as well as comparison between hours. This strategy was used in all experiments, unless otherwise stated.

#### B) Acid secretion during an infusion of fragment 1‐35 (n = 6)

Blood samples and gastric juice were collected as under A). The progastrin fragment 1‐35 was diluted in isotonic saline containing 1 g/L of human serum albumin after sterile filtration. The syringes were weighed before and after the infusions and the remaining peptide solution from the infusion was stored at −20°C until radioimmunoassay. The examination began with a 60 min control period during which isotonic saline was infused intravenously at 20 mL/h. The progastrin fragment 1‐35 was hereafter infused in three consecutive dose rates of 40, 80, and 300 pmol/kg/h; each period lasting 60 min.

#### C) Acid secretion during an increasing infusion of progastrin fragment 1‐35 and a constant gastrin‐17 infusion (*n* = 9)

Gastrin‐17 was infused in a constant rate of 40 pmol/kg/h during the whole experiment. After a control period of 60 min during which acid output reached steady state, progastrin fragment 1‐35 was administered in three consecutive dose rates of 40, 80, and 300 pmol/kg/h with each dose period lasting 60 min.

#### D) Acid secretion during a sudden 1‐h infusion of progastrin fragment 1‐35 fragment and a constant gastrin‐17 infusion (*n* = 6)

A 1‐h basal period was allowed. Acid secretion was then stimulated for 45 min with a 20 pmol/kg/h gastrin‐17 followed by a 40 pmol/kg/h dose. After reaching steady state in acid output (45 min after the 40 pmol/kg/h dose start), the 400 pmol/kg/h progastrin fragment 1‐35 was infused for 1 h. Hereafter, only the gastrin‐17 infusion was continued for the remaining time of the experiment.

#### E) Acid secretion during a sudden 1‐h infusion of progastrin fragment 6‐35 and a constant gastrin‐17 infusion (*n* = 6)

Experimental protocol was as under D). Instead of progastrin fragment 1‐35, the progastrin fragment 6‐35 was infused at a calculated dose rate of 400 pmol/kg/h.

#### F) Acid secretion during a sudden 1‐h infusion of progastrin fragment 1‐19 and a constant gastrin‐17 infusion (*n* = 6)

Experimental protocol was as under D). Instead of progastrin fragment 1‐35, the progastrin fragment 1‐19 was infused at 300 pmol/kg/h.

*A‐F)* The recovery of gastric juice was determined by infusion of a marker in the lateral lumen of the tube (230 kBq ^57^Co‐labeled cobalamin, 1.25 mg cobalamin, and 1 g human albumin diluted in 1000 mL isotonic saline, 60 mL/h). The lateral lumen ended 12 cm proximal to the openings of the central canal. The volume of gastric juice was measured for each 15 min period, and the concentration of H^+^ was determined by titration to pH 7.0 with an autotitrator (PMH 26, Radiometer, Denmark). Blood was collected in chilled tubes containing 39 *μ*mol di‐sodium‐EDTA and immediately placed on ice. Plasma was stored at −20°C until radioimmunoassay.

### Radioimmunoassays

The following antisera were used. Antiserum 94023 was raised against the N‐terminus of human progastrin and measures the plasma concentrations of N‐terminal progastrin fragments 1‐35 and 1‐19 as described elsewhere (Goetze et al. 2006). This antiserum recognizes only the very N‐terminal pentapeptide sequence of human progastrin and displays no cross‐reactivity to other known peptides. N‐terminal progastrin 1‐10 extended C‐terminally with a tyrosyl residue was used as standards and tracer where the tracer peptide was monoiodinated for detection. For measurement of progastrin fragment 6‐35, we used antiserum 88235, which recognizes the epitope 20‐25 of human progastrin and hence binds to the N‐terminus of gastrin‐52 (Rehfeld and Johnsen 1994). Antiserum 88235 does not react with carboxyamidated gastrin‐34, ‐17 or smaller gastrins. Prior to radioimmunoassay, plasma samples were treated with trypsin for 30 min at room temperature followed by boiling for 10 min to achieve maximal binding. This procedure cleaves the 1‐35 fragment at the monobasic site at amino acid 19, thereby maximally exposing the sequence 20‐25 epitope in progastrin. Synthetic fragment 20‐32 extended with tyrosine at the C‐terminus was used as standard and the corresponding mono‐iodinated peptide was used as tracer. Finally, antiserum 2604 was used to measure gastrin‐17 (Rehfeld et al. 1972; Stadil and Rehfeld 1973). This assay specifically binds the C‐terminus common for all acid stimulatory gastrins. Hence, antiserum 2604 binds gastrin‐71, ‐34, and ‐17 with equimolar potency and its reactivity with cholecystokinin peptides is negligible. Synthetic human gastrin‐17 was used as standard and mono‐iodinated ^125^I‐gastrin‐17 as tracer (Stadil and Rehfeld 1972).

### Chromatography

Plasma samples from subjects in the separate experiments were pooled at the end of the relevant infusion periods. A 1 mL sample was then applied to a 2000 × 10 mm Sephadex G‐50 Superfine column (Pharmacia, Uppsala, Sweden) and the column was eluted with a barbital buffer containing bovine albumin (pH 8.2) at 4°C. Fractions of 2 mL were collected. Void and total volume was determined by elution of ^125^I‐albumin and ^22^NaCL, respectively. The column was earlier calibrated with synthetic progastrin fragments 1‐35, 1‐19, and 6‐35 and extracts of human antral tissue. All fractions were assayed with the radioimmunoassay described above.

### Calculations and statistical analysis

Correction for nonrecovered gastric juice was made for every 15 min during the infusion experiments (A‐F) according to the equation: V_C_ = V_A_ × Q_i_/Q_a_, where V_C_ is the corrected volume, V_A_ the apparent volume, Q_i_ the amount of radioactivity (counts per min) infused, and Q_a_ the amount of aspirated radioactivity. All results are expressed as means ± SEM. Statistical analysis was performed by Friedman's nonparametric two‐way analysis of variance and *P* < 0.05 were considered significant.

## Results

### Acid secretion increases during infusion of gastrin‐17 (part A, *n* = 6)

**Figure 1:**
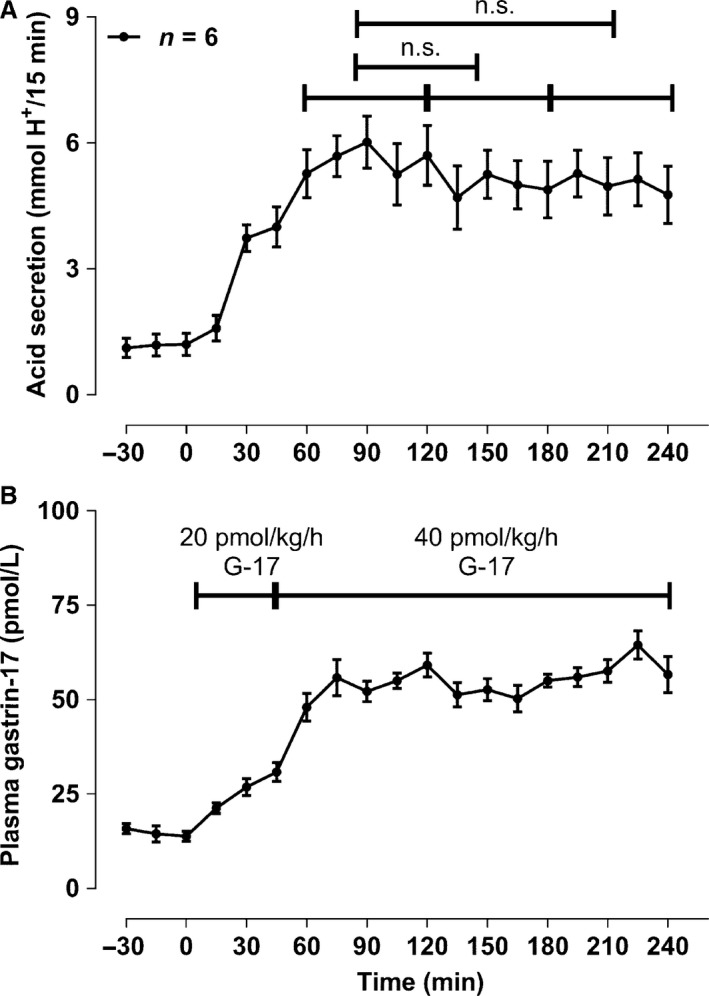
Control infusion of gastrin‐17. A singular and constant infusion of gastrin‐17 resulted in a steady state in gastric acid output during the entire time course of the examination. No fading in acid secretion was demonstrated. Data are given as means ± SEM.

The effective gastrin‐17 dose rate was 34 ± 3.9 pmol/kg/h during the 40 pmol/kg/h infusion. No decrease in the stimulated acid secretion was detected in the time span of the study, where the acid secretion had reached steady state (Fig. 1).

### Acid secretion does not change during infusion of fragment 1‐35 (part B, *n* = 6)

The effective dose rates as estimated from measurements of peptide in the infusion lines were 34.0 ± 2.5, 67.3 ± 5.5, and 295 ± 10.4 pmol/kg/h. Mean acid output was 0.55 ± 0.05 mmol H^+^/15 min in the control period, and during the three consecutive dose periods, it was 0.34 ± 0.09, 0.34 ± 0.14, and 0.31 ± 0.19 mmol H^+^/15 min. None of these dose periods was significantly different from the control period (data not shown).

### Acid secretion decreases during an increasing infusion of progastrin fragment 1‐35 and a constant gastrin‐17 infusion (part C, *n* = 9)

**Figure 2:**
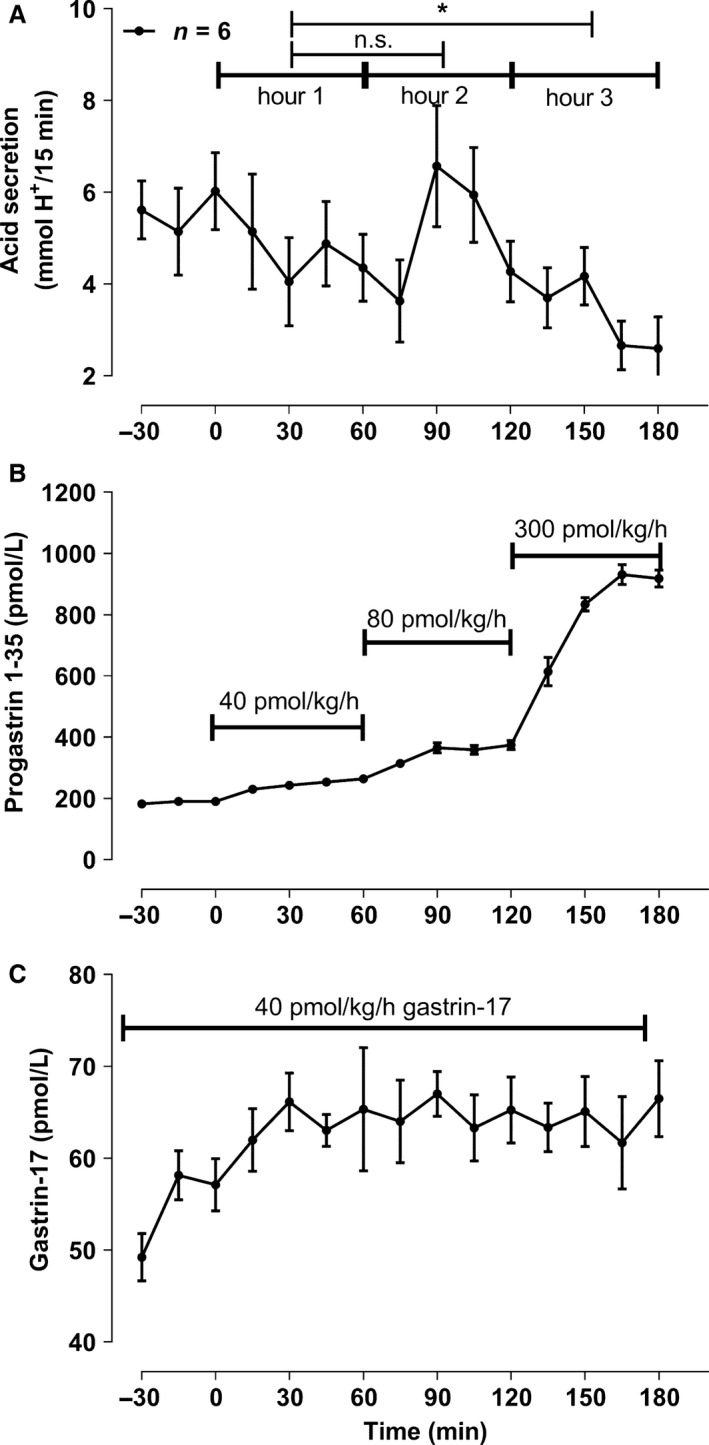
Gastric acid output during constant gastrin‐17 infusion and a stepwise increase in progastrin fragment 1‐35 infusion dose as described under C) in the methods section. The first 40 pmol/kg/h dose of progastrin fragment 1‐35 was administered during the first 60 min, then an 80 mol/kg/h infusion from 60–120 min, and finally the high 300 pmol/kg/h dose during 120–180 min. Only mean and SEM values for period – 30 min and forward are shown. n.s. denotes nonsignificant, and the * represents a P < 0.05.

The effective dose rate of gastrin‐17 was 43.3 ± 2.0 pmol/kg/h. The plasma concentration of gastrin‐17 and gastric acid secretion reached a plateau after 30 min (Fig. 2), with a mean acid output of 5.63 ± 0.73 mmol H^+^/15 min before the infusion of progastrin fragment 1‐35. The dose rates of progastrin fragment 1‐35 were 33.2 ± 2.7, 66.4 ± 5.3, and 276.8 ± 13.0 pmol/kg/h. Mean acid output during concomitant infusion of the two peptides was 4.64 ± 0.83, 5.10 ± 0.62, and 3.28 ± 0.55 mmol H^+^/15 min (Fig. 2). Only the mean acid output during the last dose interval was significantly lower than the acid output during the control period (*P* < 0.05) with a 29% decrease in acid secretion. Mean acid output in the last dose interval was also significantly lower when compared with the other dose intervals (*P* < 0.05).

### Acid secretion decreases after a sudden 1‐h infusion of progastrin fragment 1‐35 fragment and a constant gastrin‐17 infusion (part D, *n* = 6)

**Figure 3:**
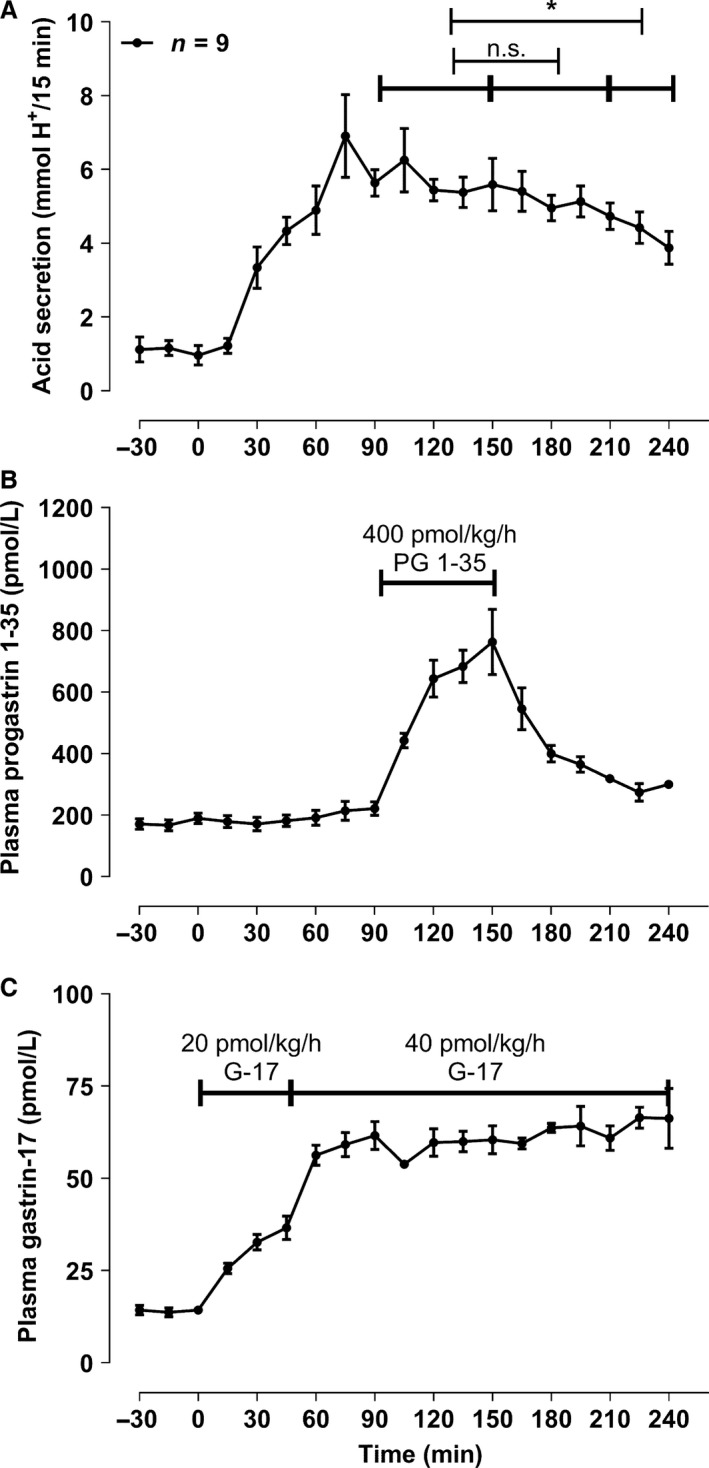
Constant gastrin‐17 infusion and a sudden 1‐h (t_90‐150_) 400 pmol/kg/h infusion of progastrin fragment 1‐35 as described under D) in methods. Only mean and SEM values for period – 30 min and forward are shown. n.s. denotes nonsignificant, and the * represents a P < 0.05.

The constant gastrin‐17 dose rate was 45 ± 2.8 pmol/kg/h and the progastrin fragment 1‐35 dose given in the 1‐h period was 380 ± 13 pmol/kg/h. A decrease in the mean acid output was not observed during the 1‐h infusion of progastrin fragment 1‐35 (Fig. 3). A decrease in acid output was, however, noted during the last period of the examination. The plasma concentration of gastrin‐17 was constant during the whole infusion.

### Acid secretion does not change during a sudden 1‐h infusion of progastrin fragment 6‐35 and a constant gastrin‐17 infusion (part E, *n* = 6)

**Figure 4:**
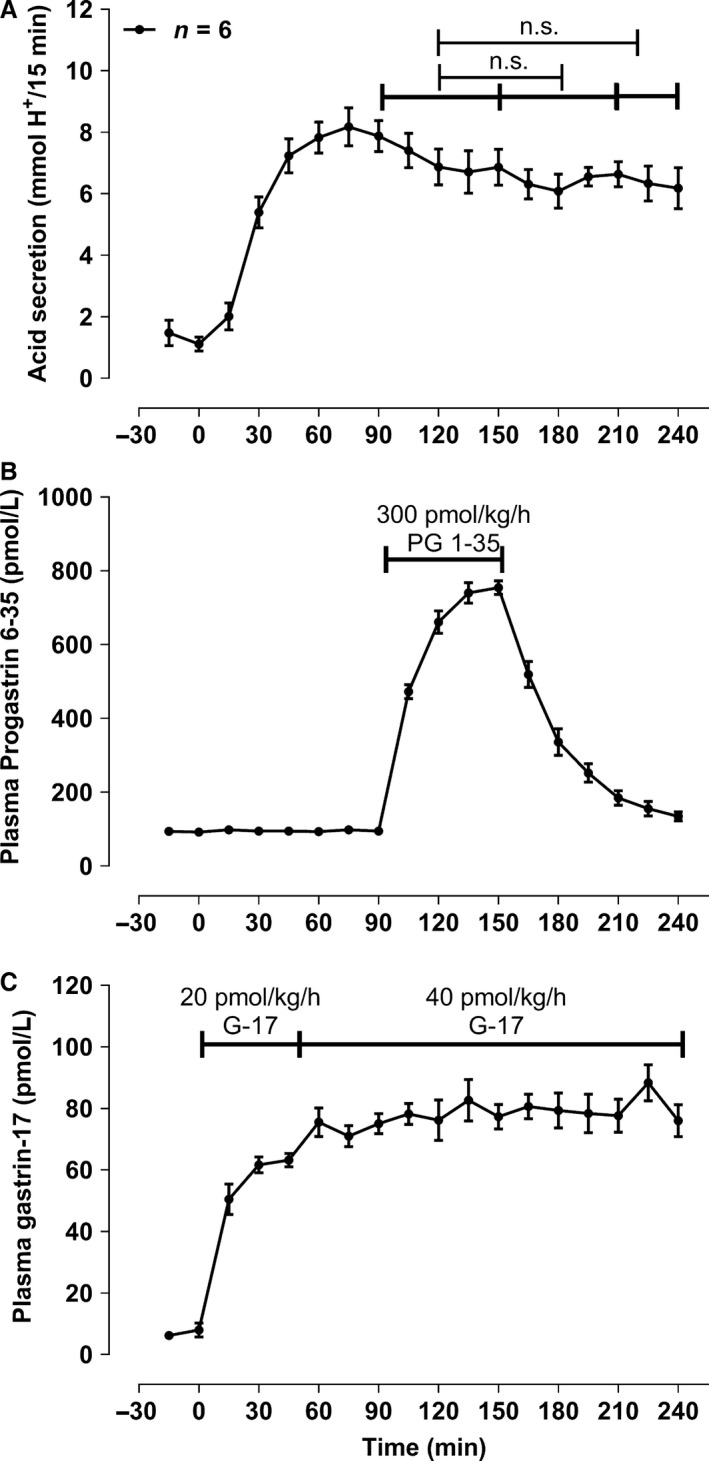
Constant gastrin‐17 infusion and a sudden 1‐h (t_90‐150_) 400 pmol/kg/h infusion of the progastrin fragment 6‐35 as described under E) in methods. Only mean and SEM values for period – 30 min and forward are shown. n.s. denotes nonsignificant, and the * represents a P < 0.05.

The administered gastrin‐17 dose rate was 49.8 ± 2.2 pmol/kg/h and resulted in a stable plasma concentration after steady state was reached (Fig. 4). Progastrin fragment 6‐35 was infused in a 416 ± 5.4 pmol/kg/h rate with no significant change in acid output detected in the periods including the end period after the progastrin fragment 6‐35 infusion.

### Acid secretion during a sudden 1‐h infusion of progastrin fragment 1‐19 and a constant gastrin‐17 infusion (part F, *n* = 6)

**Figure 5:**
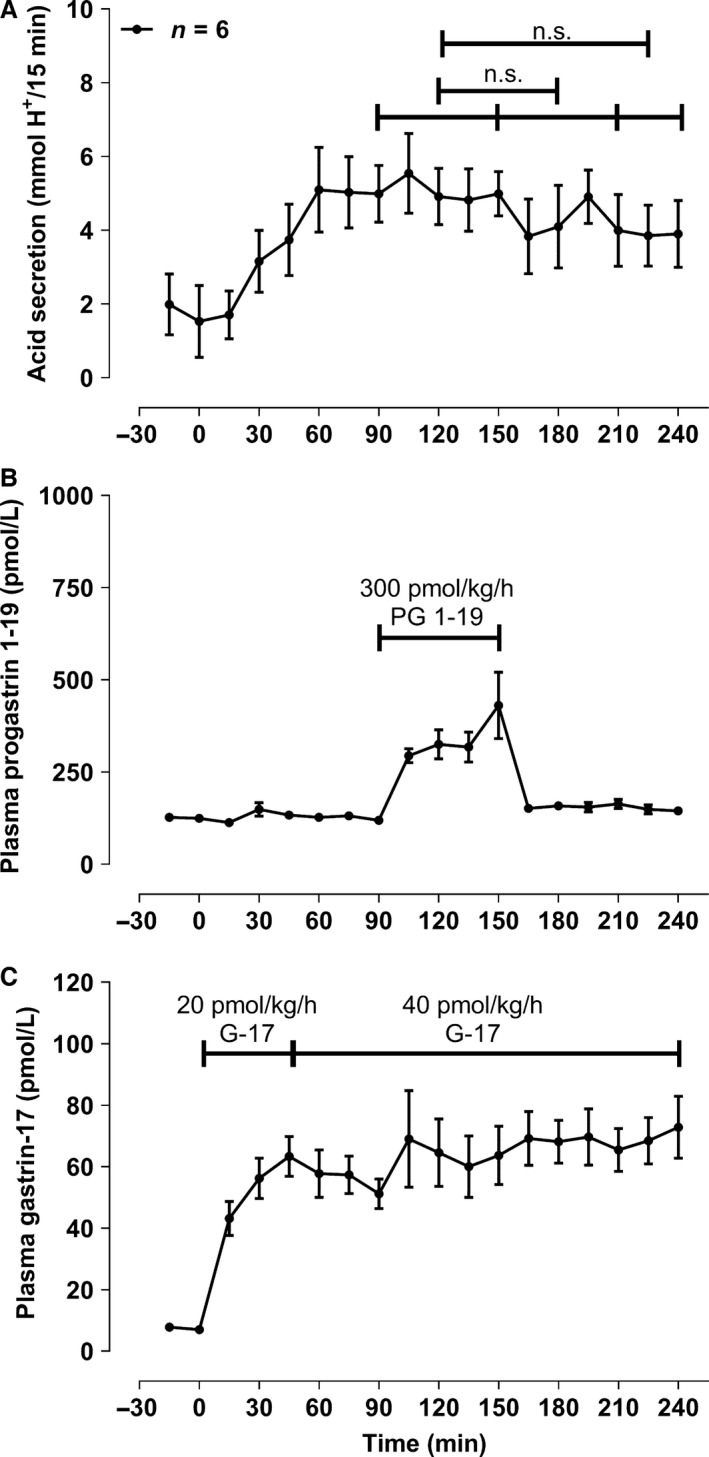
Constant gastrin‐17 and a sudden 1‐h (t_90‐150_) 300 pmol/kg/h infusion of the progastrin fragment 1‐19 as described under F) in methods. Only mean and SEM values for period – 30 min and forward are shown. n.s. denotes nonsignificant, and the * represents a P < 0.05.

The effective gastrin‐17 dose rate was 61 ± 6.1 pmol/kg/h and progastrin fragment 1‐19 was infused for 1 h in a 268 ± 28.5 pmol/kg/h dose. A maximal fragment 1‐19 plasma concentration of 430.5 ± 89.9 pmol/L was reached at the end of the infusion (t_150_, Fig. 5). This concentration of the 1‐19 fragment was half of that of the longer progastrin fragment 1‐35 in experiment D). Gastric acid secretion was only moderately decreased during the infusion period (t_90‐150_) with no significant decrease in acid output in the final period of the experiment.

### Chromatography

Elution of plasma samples from the infusion of progastrin fragment 1‐35 and gastrin‐17 in experiment C) at the end (*t* = 180 min, Fig. 3) revealed only one immunoreactive peak eluting in the same position as the calibration peptide of N‐terminal progastrin fragment 1‐35 (K_D_ = 0.58, data not shown). No other peaks were detected, suggesting that the infused peptide was not cleaved to smaller N‐terminal fragments during the time course of the examination.

## Discussion

Novel roles of gastrins, including progastrin, are currently under discussion (Giraud et al. 2016; Rehfeld 2016). This study suggests that N‐terminal progastrin fragments infused in high doses can acutely inhibit gastrin‐stimulated acid secretion in man. The inhibitory effect was, however, only observed when the fragments were administered in high doses and there was no clear dose–response relationship. In contrast, the inhibitory effect could not be achieved with an equimolar dose infusion of the N‐terminally truncated form: progastrin 6‐35. The peptide mediating the effect hence seems to require an intact N‐terminus of progastrin.

Acid stimulatory gastrins are peptides of different chain length which all have the C‐terminal tetrapeptide amide Trp‐Met‐Asp‐Phe‐NH_2_ as active site (Gregory and Tracy 1964; Morley et al. 1965; Hilsted and Rehfeld 1987). Also, gastrin peptides extended C‐terminally with glycine have been suggested to exert unique biological effects (Seva et al. 1994). Glycine‐extended gastrins have earlier been examined for their possible role in gastric acid secretion. However, we could not detect any effect on acid stimulation using the same setup as the present one – even when administering the peptide in supraphysiological concentrations (Hansen et al. 1996; Hilsted et al. 1988; for review, see Rehfeld 2014). Other progastrin‐derived peptides have also been examined for biological activity (Wang et al. 1996; Baldwin et al. 2001; Smith et al. 2006). Some of these studies, however, did not evaluate the biological responses to a specific region of the precursor. One study examined a recombinant N‐terminal truncated form of the precursor, that is*,* progastrin 6‐80, thus excluding the N‐terminal sequence 1‐5 (Baldwin et al. 2001). As this specific epitope was necessary for effect in our study, it seems, at present, not straightforward to combine the biological connotation of the findings. On the other hand, we cannot rule out that the N‐terminus enhances the effects but still depends on the remaining peptide structure.

N‐terminal progastrins circulate in humans in an almost 30‐fold higher concentration than the C‐terminal gastrins during fasting states (Goetze et al. 2006). In this study, we also showed that meal stimulation increases both N‐terminal progastrin fragments and amidated gastrins with gastrin concentrations reaching a plateau 30 min after stimulation. The concentration of N‐terminal progastrin fragments, however, remained elevated throughout the entire time period studied after stimulation. Moreover, we have shown the N‐terminal fragments to be released in a more constitutive manner, rather than the well‐known regulated secretion pattern of the C‐terminal gastrins (Bundgaard and Rehfeld 2008). This suggests that a relevant biological situation for the inhibitory effect may be during the fasting states when gastrin concentrations are low. According to such a theory, the acid inhibition of N‐terminal progastrins during stimulated gastrin release, for example, during meals, would yield to the brief C‐terminal gastrin stimulation of acid secretion. The present observed inhibitory effect might therefore have been studied under suboptimal physiological conditions, as inhibition of acid secretion was evaluated during a strong and constant stimulation with gastrin‐17. This could also partly explain the lack of a clear dose–response relationship. Why the effect is seen after the infusion is not well explained but may suggest that the effect requires intracellular changes, as for instance transcriptional regulation, before the effect on acid secretion precipitates. In extension of this, it should also be noted that any potential long‐term effects are not addressed in this study protocol. Therefore, studies addressing long‐term effects on cellular number/growth and differentiation needs to be pursued.

The acid secretory response from the parietal cells to gastrin is primarily mediated through the histamine‐releasing endocrine‐like cells – the ECL cells. The response to N‐terminal progastrin fragments may, on the other hand, be mediated only through the parietal cell, and thus the inhibitory effect would not be biologically amplified in the same manner as for the C‐terminal gastrins. This fits with the different potencies of N‐terminal progastrins and C‐terminal gastrins. Also, the somatostatin‐producing D‐cells should be considered as mediator of the effect. A stimulation of paracrine somatostatin response would regulate both the histamine release and the acid secretion during fasting states.

In conclusion, this study suggests that the N‐terminal region of human progastrin can inhibit gastrin acid secretion in man. The inhibitory effect was diminished when removing the N‐terminal pentapeptide sequence, which indicates that this region is involved in the inhibitory effect. For now, we suggest that N‐terminal prograstrin fragments may play a role for keeping gastric acid secretion low between meals.

## Conflict of Interest

The authors have no conflicts of interest.
